# Improved visualisation of early cerebral infarctions after endovascular stroke therapy using dual-energy computed tomography oedema maps

**DOI:** 10.1007/s00330-018-5449-4

**Published:** 2018-05-04

**Authors:** Astrid Ellen Grams, Tanja Djurdjevic, Rafael Rehwald, Thomas Schiestl, Florian Dazinger, Ruth Steiger, Michael Knoflach, Elke Ruth Gizewski, Bernhard Glodny

**Affiliations:** 1 Department of Neuroradiology, Medical University of Innsbruck, Anichstraße 35, 6020 Innsbruck, Austria; 2 Institute of Neurology, University College London, London, UK; 3 Department of Radiology, University of Cambridge, Cambridge, UK; 4 Department of Neurology, Medical University of Innsbruck, Innsbruck, Austria; 5 Department of Radiology, Medical University of Innsbruck, Innsbruck, Austria

**Keywords:** Dual-energy CT, Cerebral infarction, Computed tomography, Stroke, Thrombectomy

## Abstract

**Objective:**

The aim was to investigate whether dual-energy computed tomography (DECT) reconstructions optimised for oedema visualisation (oedema map; EM) facilitate an improved detection of early infarctions after endovascular stroke therapy (EST).

**Methods:**

Forty-six patients (21 women; 25 men; mean age: 63 years; range 24–89 years) were included. The brain window (BW), virtual non-contrast (VNC) and modified VNC series based on a three-material decomposition technique optimised for oedema visualisation (EM) were evaluated. Follow-up imaging was used as the standard for comparison. Contralateral side to infarction differences in density (CIDs) were determined. Infarction detectability was assessed by two blinded readers, as well as image noise and contrast using Likert scales. ROC analyses were performed and the respective Youden indices calculated for cut-off analysis.

**Results:**

The highest CIDs were found in the EM series (73.3 ± 49.3 HU), compared with the BW (-1.72 ± 13.29 HU) and the VNC (8.30 ± 4.74 HU) series. The EM was found to have the highest infarction detection rates (area under the curve: 0.97 vs. 0.54 and 0.90, *p* < 0.01) with a cut-off value of < 50.7 HU, despite slightly more pronounced image noise. The location of the infarction did not affect detectability (*p* > 0.05 each).

**Conclusions:**

The EM series allows higher contrast and better early infarction detection than the VNC or BW series after EST.

**Key Points:**

*• Dual-energy CT EM allows better early infarction detection than standard brain window.*

*• Dual-energy CT EM series allow better early infarction detection than VNC series.*

*• Dual-energy CT EM are modified VNC based on water content of tissue.*

## Introduction

Endovascular stroke treatment (EST) is the method of choice for acute stroke patients with proximal intracerebral vessel occlusion, with or without prior lysis. A computer tomography (CT) scan must first be conducted to rule out an intracerebral haemorrhage (IH) [1] or determine the ASPECTS score [2] if appropriate. In addition, however, due to the need for post-interventional antiaggregation therapy, it is crucial to assess infarction size and to rule out IH immediately after EST as well. Using CT after EST, hyperdense IH cannot be differentiated from hyperdense blood-brain barrier (BBB) disruption. Both may mask an underlying developing infarction [3]. However, DECT can reliably distinguish IH from contrast agent (CA) due to BBB disruption after administration of CA [4–7], but it could also be useful for the early visualisation of the infarction prior to EST [8, 9].

Beginning with the early onset of the infarction, glucose, dioxygen and ATP are consumed, electrochemical gradient loss occurs, the cell depolarizes and a cytotoxic oedema develops [10]. Vascular endothelial cells are damaged, triggering the formation of a ionic oedema [11]. Na^+^ is replenished in the extracellular space and water follows the osmotic gradient. Endothelial protein leakage marks the beginning of vasogenic oedema formation and the breakdown of the BBB [11]. The accumulation of water is probably the phenomenon detected by the DECT [8, 9].

The DECT technology is based on the light-matter interactions that are dependent on the material and the radiation energy [12]. Depending on the binding energy of K-shell electrons, which are different in different substances, absorption peaks occur. They correspond to specific energies at which X-ray absorption increases for specific substances, i.e. for iodine at 32.2 keV [12–14]. Due to the different absorption characteristics of different substances with high and low atomic numbers at different X-ray energies, the contribution of the high-density material to the total absorption of a mixture of three materials – two with low attenuation and one with higher attenuation – can be determined [12]. This is known as the three-material decomposition technique (TMDT), which allows for the reconstruction of a blended image, a so-called material map – in the case of CA, an iodine map (IM), or using subtraction, a virtual non-contrast (VNC) map [6, 12, 15, 16]. The first potential application of this technique is to quantify a component in a three-component mixture [14], and consists of differentiating between iodine and blood [4–6], detection of pulmonary embolism or myocardial ischaemia, atherosclerotic plaque removal [14], imaging of gout lesions [17] or metal artefact reduction [17]. The second main application of DECT is for classifying materials. The best-known example is discriminating between uric acid and non-uric acid stones [14].

Recently, new applications for DECT-derived TMDT have been published, aiming at the visualisation of brain oedema to detect infarctions as early as possible [8, 9]. These are modified VNC series [8] or bone marrow oedema series, respectively, called ‘X map’ [9]. The technique is based on the assumption that the brain mainly consists of white matter (WM), grey matter (GM) and water, that the difference in absorption between WM and GM is due to the difference in lipid content, and that infarctions – irrespective of their pathogenetic mechanism – accumulate a relatively high amount of water during their development. Subtracting the lipid content from the WM results in a GM and water content map that should make infarctions easier to visualise [9]. The method described was developed in parallel at our institution with minor modifications.

The objective of this study was to evaluate whether the modified GM and water content map, called the ‘oedema map’, allows better detection of early cerebral infarctions than the standard VNC or BW series after EST.

## Materials and methods

This retrospective study was approved by the local institutional ethical review board (IRB AN2014-0158 337/4.6). Individual informed consent was waived by the IRB. DECTs have been performed routinely after EST since 2013 to detect BBB disruptions and IH [18]. Forty-six patients (21 women, 25 men; mean age: 63 years (range: 24–89 years) who received CT immediately after admission [1, 2] and who had EST and immediate post-interventional DECT were included. Only the DECTs performed between October 2013 and January 2016 immediately after EST were included in this study; the non-contrast CTs prior to EST were not performed as DECT and consequently not included. The average time from the onset of stroke symptoms to DECT time was 6 ± 2 h. In all patients, mechanical endovascular thrombectomy using a stent retriever was performed within this time frame (34 due to a middle cerebral artery occlusion; 12 due to a basilar artery occlusion).

### Dual-energy computed tomography

A DECT scan (Somatom Definition Flash, Siemens Healthcare, Erlangen, Germany) of the brain was performed within no more than 1 h after EST in all patients. The first tube was operated at 100 kV and 360 mA, the second tube at 140 kV and 360 mA using a selenium filter and beam-hardening correction, respectively. The pitch factor was 0.45, the acquisition mode was incremental with a 32 x 0.6 mm collimation, the slice thickness was 4 mm, the image kernel was H30f medium smooth and the scan field of view was 200 mm.

### Image reconstruction

All secondary reconstruction images had the same axial alignment as the source images, a display field-of-view of 200 mm, and an image matrix of 512 x 512 pixels (EM and BW series slice thickness: 4 mm; increment: 4 mm; kernel: H30f medium smooth; window: base orbit; standard VNC series slice thickness: 1 mm, increment: 1 mm). The reconstructions were performed at a dedicated post-processing workstation (Brain Hemorrhage; SyngoCT workplace 2012B, Siemens Healthcare, Erlangen, Germany).

**Fig. 1:**
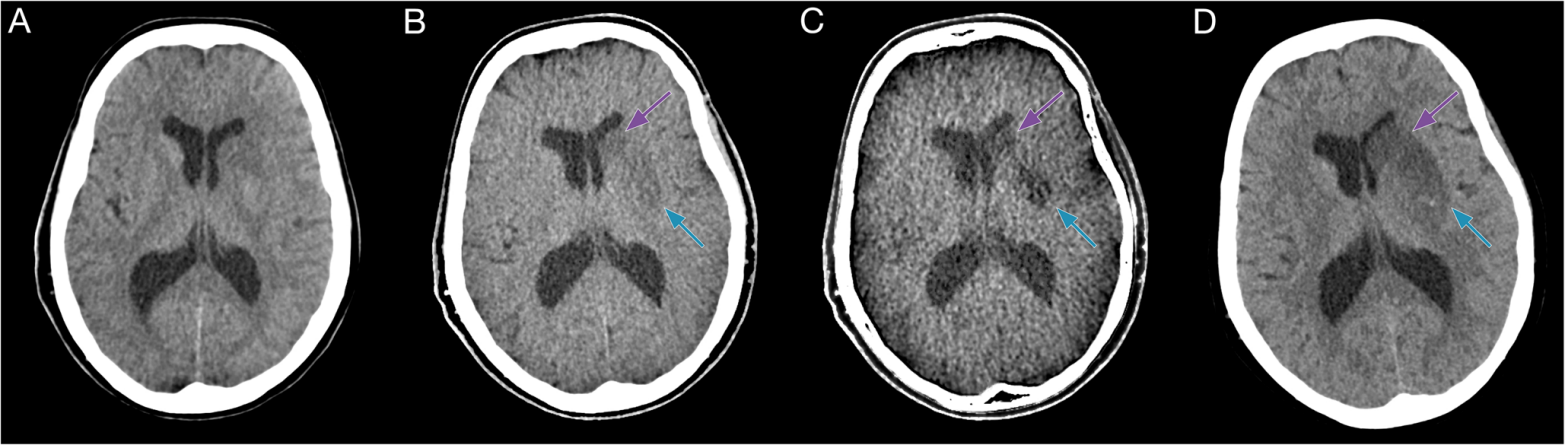
Example of ischaemic lesions in the caudate nucleus (violet arrow) and putamen (blue arrow) in the BW (**A**), VNC (**B**) and EM (**C**) series. In the BW (**A**) and VNC (**B**) series, the lesions are not clearly visible, however in the EM the hypodense infarctions can be clearly identified. Follow-up CT with infarction delineation (**D**)

The settings for the EM were determined based on a sample of 18 patients. Test data for the determination of the best reconstruction settings was generated by one author (AEG) and submitted to another (GB) for assessment. The EM is an imaging method for visualising GM and water content that was generated under the assumption that the brain consists of GM, WM and water [9]. Because GM contains 8% more oxygen, but 8% less carbon than WM, its photoelectric absorption is higher than that of WM [9]. The density of GM in the 18 patients was 38.7 ± 2.7 HU at 100 kV and 31.4 ± 3.1 HU at Sn140 kV, while the density of the WM was 29.7 ± 1.8 at 100 kV and 27.1 ± 0.6 HU at Sn140 kV. Since the standard deviation was less than 10% of the measured values themselves, we did not measure the values in all 46 patients. The densities of the infarctions determined with knowledge of the later development (observer: AEG) were 29.5 ± 3.8 HU at 100 kV and 25.8 ± 3.7 HU at Sn140 kV. They were similar to the WM densities. Ultimately, 39/31 HU (100 kV/Sn140 kV) was selected as the setting to generate the EM for the first component, and 30/26 HU for the second component. We chose a slope of 1.3 with which the subjectively best visualisation of the infarction was achieved. The applied slope was evaluated in preliminarily tests, in which imaging data with a slope between 1.1 and 2.0 was generated in 0.1 increments (AEG) and randomly assessed by one independent observer (BG) for infarction detectability. The relation corresponds to the ratio of the GM densities 100 kV versus Sn140 kV. Since the result was convincing, the slope of 1.3 was set as standard and was not changed for this study, the objective of which was in principle to prove superior infarction detection with the EM series after EST. The resulting optimised EM series for infarction visualisation (Fig. 1) compensates for the differences between GM and WM, comparable with Noguchi et al. [9]. The EM series has subjectively higher noise than the source series and tends to feature slightly lower densities in the periphery of the brain adjacent to the calvaria; however, the overall quality is good. Iodine was not completely suppressed in the series.

### Image evaluation

The subjective detectability of the infarctions of all patients in the EM and the other series was evaluated by two experienced, blinded raters (MK and TD). The imaging data were presented in random order. The visible contrast between the infarctions and the adjacent brain (1 = good contrast, 2 = moderate contrast, 3 = little contrast, 4 = no contrast) and the presence of image noise (1 = no noise, 2 = little noise with no disturbance of the infarction evaluation, 3 = moderate noise with little disturbance of the infarction evaluation, 4 = severe noise with marked disturbance of the infarction evaluation) were visually evaluated using four-point Likert scales [19, 20].

### Follow-up image evaluation

**Fig. 2:**
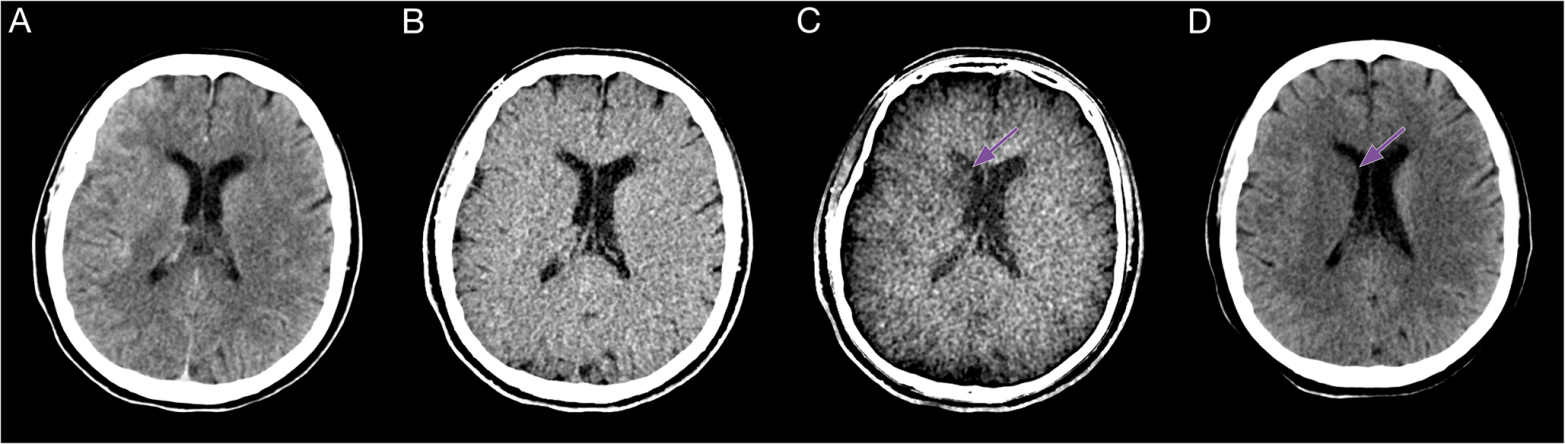
Example of a caudate nucleus infarction in the BW (**A**), VNC (**B**) and EM (**C**) series. On the EM, the infarction displays pronounced contrast relative to nearby brain parenchyma (arrow). Follow-up CT with infarction delineation (**D**, arrow)

The follow-up (FU) image evaluation was performed by two experienced neuroradiologists (AEG and TD). Infarctions were assessed by analysing FU imaging (Figs. 1 and 2), performed within a mean of 24 ± 4 h after treatment, which either was a standard CT (n = 32) or an MRI with diffusion- and susceptibility-weighted imaging (SWI) sequences (n = 14). With knowledge of the identified infarctions, density region-of-interest (ROI) measurements were performed on the different reconstructed DE series using the in-house picture archiving and communication system (PACS; IMPAX EE R20 XIV v2014, Agfa, Mortsel, Belgium) and a dedicated workstation. Three ROIs (diameter range: 5–10 mm) were placed in one (up to four) distinct infarction area per patient and in the corresponding contralateral areas. To avoid displacement, ROIs were positioned on the BW series by comparing the FU series and copied electronically on all other series. From the mean tissue densities, contralateral side to infarction differences (CIDs) were calculated for each series as an objective marker for the contrast between the two tissues. Infarction volumes in all reconstructed DE series and the follow-up imaging were measured using a 3D post-processing workstation (Advantage Workstation 4.6/VolumeShare 5; General Electric Company; Fairfield, CT, USA) by two experienced neuroradiologists (TD and BG). To do this, the series was digitally transferred to the workstation. The infarction was circumnavigated in axial planes using a marking tool. Once marked in all slices, the object was cut out throughout all slices by a ‘scissors tool’, keeping the selected parts of the volume. After this, another tool can be applied to determine the volume of the selected infarction.

### Statistical analysis

The statistics for this study were calculated using GraphPad Prism, Version 7 (GraphPad Software Inc., La Jolla, CA, USA) and SPSS 23 (IBM SPSS statistics, Armonk, NY, USA). Normal distribution was not assumed. Groups of continuous data were either compared using the Mann-Whitney test or the Kruskal-Wallis test followed by Dunn’s post-hoc test. Groups of categorical variables were compared using the chi-square test. Receiver operating curve (ROC) analyses were used to determine sensitivities and specificities of each series. The cut-off values were calculated by a peak value analysis of the Youden indices derived from the ROC graph [21]. The inter-rater variability between the two raters was either determined by using the intraclass correlation coefficient (ICC) for continuous data or Cohen’s kappa coefficient for ordinal data. Statistical significance was assumed at *p* < 0.05.

## Results

Descriptive data are presented in Table 1. Of the overall 110 identified individual infarctions, 95 were found to be supratentorial (46 deep; 49 superficial) and 15 were infratentorial: seven in the thalamus, 24 in the putamen, 14 in the caudate nucleus, four in the pons, 12 in the cerebellum, 19 in the insula, eight in the occipital-, six in the frontal-, 11 in the temporal- and ten in the parietal lobe.

**Table 1:** Summary statistics of the image evaluation in the brain window (BW), virtual non-contrast (VNC) and oedema map (EM) series as well as in the follow-up imaging (FU). Overall means and standard deviations (x̅ ± σ), as well as corresponding inter-rater correlations (ICC), from the infarction density measurements, the contralateral side measurements, and the infarction to contralateral side differences (CIDs) in Hounsfield units (HU), respectively, with significance level (MW, Mann-Whitney test). The infarction volume is reported in milliliters (ml)

		Infarction density (HU)	Contralateral density (HU)	CID (HU)	Infarction volume (ml)
BW	x̅ ± σ	37.49 ± 13.75	35.64 ± 3.78	-1.72 ± 13.29	8.90 ± 0.92
	ICC	0.86	0.82	-	0.97
	*MW*	*p = 0.36*			
VNC	x̅ ± σ	25.44 ± 5.51	33.75 ± 3.23	8.30 ± 4.74	9.86 ± 1.39
	ICC	0.53	0.74	-	0.96
	*MW*	*p < 0.01*			
EM	x̅ ± σ	1.64 ± 45.25	75.48 ± 18.64	73.34 ± 49.32	22.28 ± 3.11
	ICC	0.82	0.87	-	0.83
	*MW*	*p < 0.01*			
FU	x̅ ± σ	-	-	-	32.43 ± 16.01
	ICC	-	-		0.62
	*MW*	*-*			

The lowest infarction densities were found in the EM series (1.6 ± 14.1 HU), followed by the VNC series (25.4 ± 3.1 HU), and the BW series (37.5 ± 3.5 HU). The highest CIDs were displayed with the EM series (73.3 ± 49.3 HU), higher than with the VNC series (8.3 ± 4.7 HU) and the BW series (-1.72 ± 13.3 HU). In the EM series, the peripheral infarction density (8.67 ± 26.13 HU) was higher than the infratentorial infarction density (-2.72 ± 50.07 HU) and density of the central infarctions (-4.44 ± 57.59 HU; *p* = 0.628). The inter-rater agreement of the density measurements and infarction volumetrics was excellent (Table 1).

Rater 1 identified 99 (90.0%) and rater 2 identified 100 (90.9%) of the overall 110 infarctions present in the EM series in the early stage after endovascular therapy, significantly more than using the VNC or BW series (chi-square test, *p* < 0.01). The detectability of the infarctions was statistically independent from the localisation of the infarction: rater 1 identified 44 of 46 deep cerebral infarctions and 44 of 49 superficial infarctions in the EM series; rater 2 identified 45 of 46 deep cerebral infarctions and 44 of 49 superficial infarctions in the EM series. Furthermore, rater 1 identified 88 of the 95 supratentorial and 11 of 15 infratentorial infarctions in the EM series; rater 2 identified 89 of 95 of supratentorial and 11 of 15 infratentorial infarctions in the EM series.

**Fig. 3:**
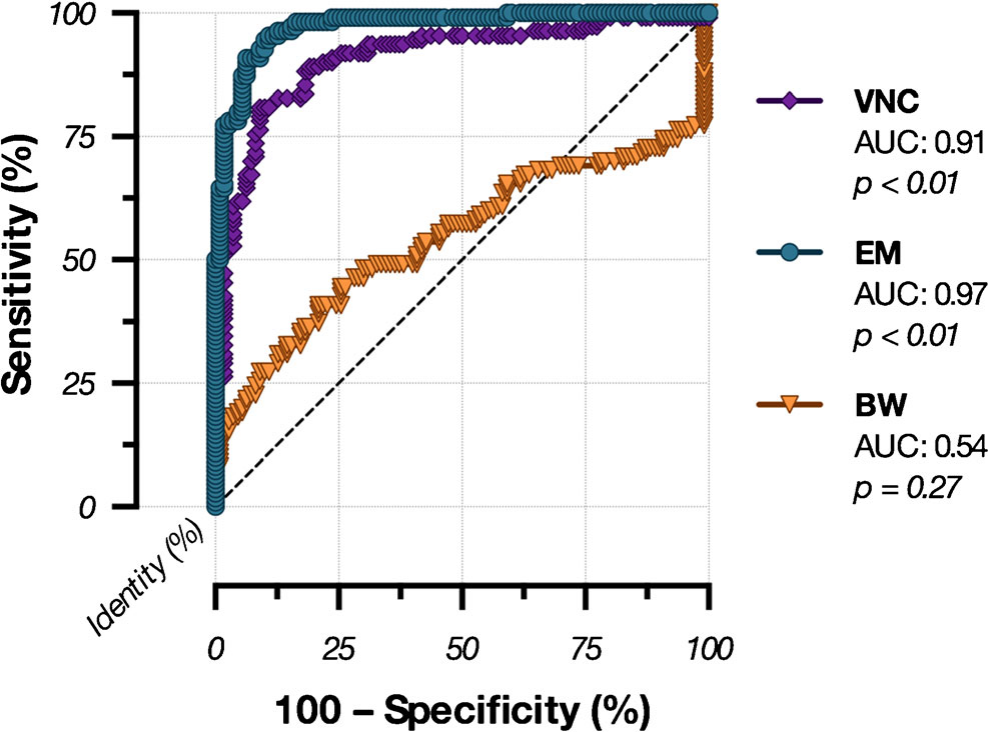
Receiver operating curves (ROC) of the subjective overall infarction detectability in the brain window (BW), virtual non-contrast (VNC), and oedema maps (EM) series. The largest area under the curve (AUC) was found in the EM series

In the ROC analysis of the infarction detectability, the EM series revealed the largest area under the curve (AUC = 0.97; *p* < 0.01), the highest sensitivity (90.91) and specificity (93.64) as well as an Youden index of 0.84 with a cut-off value of < 50.70 HU, followed by the VNC series and the BW series followed by the VNC series (AUC = 0.91, *p* < 0.01; sensitivity = 80.91; specificity = 90.91, Youden index = 0.72) and the BW series (AUC = 0.54, *p* = 0.27; sensitivity = 40.00; specificity = 79.09; Youden index = 0.19) (Table 2; Fig. 3).

**Table 2:** Sensitivity and specificity analysis with 95% confidence interval (CI), likelihood ratio and Youden index for infarction detectability in the brain window (BW), virtual non-contrast (VNC) and oedema map (EM) series

		Sensitivity [95% CI] (%)	Specificity [95% CI] (%)	Likelihood ratio	Youden index
Infarction detection	BW	40.00 [30.78–49.78]	79.09 [70.30–86.26]	1.913	0.19
	VNC	80.91 [72.31–87.78]	90.91 [83.92–95.55]	8.90	0.72
	EM	90.91 [83.92–95.55]	93.64 [87.33–97.40]	14.29	0.85

In the subjective visual evaluations, the EM series revealed the significantly highest visible infarction contrast (2.30 ± 1.20; *p* < 0.01 each) but also the most severe subjective image noise (2.37 ± 0.70; *p* < 0.01 each). Infarction contrast was considerably lower in the VNC series (3.25 ± 0.85) and lowest in the BW series (3.57 ± 0.70; *p* = 0.02). The least pronounced image noise was found in the BW series (1.21 ± 0.51), with only slightly lower values in the VNC (1.28 ± 0.55; *p* = 1.00; Table 3). Haemorrhagic transformations developed in four of the infarctions (3.6%; 9% of patients) – in one case in the right insula, one in the right putamen, one in the left thalamus and one in the left caudate nucleus.

**Table 3:** Overall visual ratings of infarction contrast and image noise in the brain window (BW), virtual non-contrast (VNC) and oedema map (EM) series. Mean values and standard deviations (x̅ ± σ), as well as Cohen’s kappa (κ) are shown

		Infarction contrast	Image noise
BW	x̅ ± σ	3.57 ± 0.70	1.21 ± 0.51
	κ	0.60	0.48
VNC	x̅ ± σ	3.25 ± 0.85	1.28 ± 0.55
	κ	0.53	0.59
EM	x̅ ± σ	2.30 ± 1.20	2.37 ± 0.70
	κ	0.47	0.53

## Discussion

In this study, modified VNC series were reconstructed from the data sets of DECT examinations immediately after EST, which enabled better visualisation of early cerebral infarctions than a standard BW or VNC series. The series was called the ‘oedema map’ (EM) and it can be described as a modified GM-water map [9] that is generated by TMDT and was first used to detect infarctions after EST. The three ‘materials’ are GM, WM and water, with partial subtraction of the lipid content. The EM series displayed significantly lower objective density values of the infarctions and higher density differences between infarctions and the respective contralateral areas, presumed to represent the detectability of an infarction, than the other evaluated series. The subjective detectability of the infarctions with the EM series demonstrated almost perfect sensitivity, specificity and a large AUC, and was thus superior to the other series despite having the highest image noise. The reproducibility between the raters was high.

However, there are some limitations of this study. First, the retrospective design has to be considered. In addition, the EM series suffered from darkening image distortions near the skull. However, despite these edge-darkening artefacts, the subjectively high image noise and the objective difference in the infarction densities depending on the infarction location, the infarction detection rates were independent of peripheral or central and supratentorial or infratentorial location. Ninety-nine and 100 of the ultimately 110 detectable infarctions were identified in the EM series by raters 1 and 2, respectively, considerably more than in the other investigated series (BW 30_R1_/30_R2_ and VNC 39_R1_/40_R2_ out of 110). The four-point Likert scales were selected to avoid indifferent evaluations by the raters and urged them to make either a positive or negative judgement. They are similar to other scales used earlier [19, 20], but have not yet been used previously in this exact form. Their functionality is assumed; however, potential effects of unintentional bias due to the scaling chosen have not been tested. Another limitation is that the optimal settings for the EM series were determined based on a sub-sample of 18 of the 46 patients included. Though the standard deviations of the measured values were low, determining the values using all datasets might have only led to slightly different settings. The modification of the slope of the WM-GM connecting line from the lipid slope of 2 [9], the theoretical target slope without the presence of CA, led to a subjectively optimised visualisation of the infarctions. The applied slope of 1.3 was chosen from a series of preliminary evaluations as the most suitable value. Whether the infarction detectability can be further optimised based on the adjustment of the reconstruction settings or whether the density differences between infarction and normal tissue, which can be generated with TMDT (EM series), can potentially be optimised if the difference of the tube voltages is increased must be determined in additional studies. Noguchi et al. and Mohammed et al. [8, 9] used comparable series for the early detection of infarctions. However, the application of these series in the early detection of infarction after EST has first been described in the present study. Imaging is more complex in this situation because the iodine-based CA adds an additional material that interferes with the optimisation of the EM series. Even the assumption of the presence of three materials [9] – GM, WM and water – is a simplification. The baseline – the connecting line between water and GM in the ‘material density diagram’ – is thus nearly identical with the line used by Noguchi et al. [9], but the slope of the connecting line between the GM and WM is smaller in order to achieve the best result. Ultimately, it is not a pure GM-water map as implemented by Noguchi et al. [9], but a modification optimised for the situation post-EST to visualise the differences between infarction and healthy brain tissue. Mean infarction densities are significantly reduced in EM series compared to both BW and VNC series because the presence of water shifts the densities of GM and WM towards lower absorption values, which then become even lower due to being projected onto the baseline [8]. The EM series appears to partially suppress CA, as the selected vector of the GM/WM connecting line partially shares a component with that of the iodine vector from the VNC map – the EM series is a modified form of a VNC map. Finally, the extent of iodine subtraction cannot be determined precisely and its applicability for detecting blood is unclear.

During a stroke, BBB disruptions often occur, much more frequently than haemorrhagic transformations. If there is BBB disruption present, the iodine CA applied during an EST leads to contrast staining in brain tissue, which can mask underlying infarctions in the BW series, but not in the VNC series [3]. In this context of CA applied, DECT is a useful tool for the differentiation of IH and contrast staining [4–7, 18, 22]. The possible superiority of a regular VNC series for the delineation of infarctions in patients after EST in comparison with a BW series has been presumed earlier [23]. However, 24 h after EST an infarction can normally be seen on a BW series in most cases. The two recent studies using DECT cited above aimed at visualising infarctions as soon as possible after their onset [8, 9]. The detection rate is comparable, with a sensitivity of 93.3% and a specificity of 100% in the study by Mohammed et al. [8], with ‘a sensitivity similar to DWI’ [9] in the study by Noguchi et al. [9] and a sensitivity of 90.91 and a specificity of 93.64 in our study – albeit it is certainly lowest in the latter because it was performed on patients after EST. The visual image impression is the same in all three studies. The noise is high, and edge-darkening artefacts are present. Since no data are available on the time from onset until detectability of infarctions with the new techniques prior [8, 9] or after EST, at this time the only hypothesis that can be proposed is that water accumulation in the infarction region is visualised using the method. Possible limitations of the detectability of infarctions, i.e. near the base of the skull or the calvaria, must be more clearly defined by future research. The use of other image kernels, or the settings of the tubes themselves, for Mohammed et al. 90 kV and 150 kV [8], for Noguchi et al. 80 kV and 150 kV [9], and in our study 100 kV and 140 kV, could provide opportunities for improvement.

In our cohort, 9% of all patients showed haemorrhagic transformation of their infarctions, as compared to 10.4%, 13.6% and 41.4% in other cohorts [6, 18, 23]. Following current guidelines, patients are treated initially with 40 mg enoxaparin-sodium twice a day and a loading dose of acetylsalicylic acid [24], but antiaggregation therapies are reduced or discontinued in the event of the new occurrence or growth of an IH.

In conclusion, the present study is the first to demonstrate that with a DECT EM map, a modified VNC map, infarctions can be reliably detected early after EST. This is crucial, as early antiaggregation treatment after thrombolysis and EST is frequently indicated, but yet harbours the risk of intracerebral haemorrhage.

## Abbreviations

ATP: Adenosine triphosphate
BBB: Blood brain barrier
BW: Brain window
CA: Contrast agent
CID: Contralateral side to infarction differences
CT: Computed tomography
DECT: Dual-energy CT
EM: Oedema map
EST: Endovascular stroke treatment
FU: Follow-up
GM: Grey matter
HU: Hounsfield units
ICC: Intraclass correlation coefficient
PACS: Picture archiving and communication system
ROC: Receiver operating curve
ROI: Region of interest
TMDT: Three-material decomposition technique
VNC: Virtual non-contrast series
WM: White matter
